# A novel frameshift variant in *UBA2* causing split-hand/foot malformations in a Pakistani family

**DOI:** 10.1038/s41439-023-00242-z

**Published:** 2023-05-23

**Authors:** Asia Parveen, Muhammad Tariq, Sher Alam Khan, Naseebullah Kakar, Amina Arif, Naveed Wasif

**Affiliations:** 1 Faculty of Science and Technology, University of Central Punjab (UCP), Lahore, Pakistan; 2 Department of Biochemistry Faculty of Life Sciences, Gulab Devi Educational Complex, Lahore, Pakistan; 3 Department of Medical Laboratory Technology, University College of Duba, University of Tabuk, Tabuk, Kingdom of Saudi Arabia; 4 Department of Biotechnology and Genetic Engineering, Kohat University of Science and Technology (KUST), Kohat, Pakistan; 5 Department of Biotechnology, BUITEMS, Quetta, Pakistan; 6 Institute of Human Genetics, University Hospital Schleswig-Holstein, Luebeck, Germany; 7 Institute of Human Genetics, Ulm University and Ulm University Medical Center, Ulm, Germany; 8 Institute of Human Genetics, University Hospital Schleswig-Holstein, Campus Kiel, Kiel, Germany

**Keywords:** Clinical genetics, Genetics

## Abstract

Split-hand/foot malformation (SHFM) shows diverse heterogeneity and manifests with reduced penetrance and variable expressivity. This study investigated the underlying genetic cause of a family segregating SHFM. Exome sequencing followed by Sanger sequencing identified a novel single nucleotide heterozygous variant (NC_000019.9 (NM_005499.3):c.1118del) in *UBA2* cosegregating in the family in an autosomal dominant manner. Our findings conclude that reduced penetrance and variable expressivity are the two remarkable and unusual features of SHFM.

## Data Report

Split-hand/foot malformation (SHFM; OMIM: 183600) or ectrodactyly is a rare congenital disorder that comprises a broad spectrum of limb abnormalities with variable phenotypes^1^. SHFM demonstrates a significant amount of clinical heterogeneity, ranging from a mild phenotype in the form of the shortening of a single central digit to a severe phenotype of monodactyly, and displays various modes of inheritance with variable expressivity and reduced penetrance^2,3^.

**Fig. 1:**
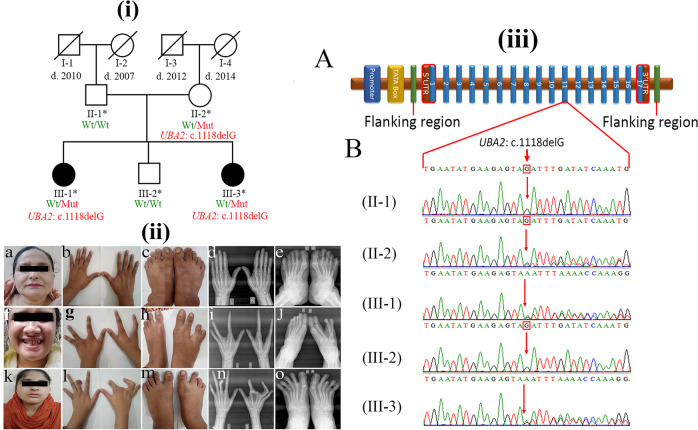
Pedigree diagram, clinical presentation, and Sanger sequencing of the family. (**i**) Pedigree diagram showing the segregation of the *UBA2* variant in an autosomal dominant manner. Male and female members are shown by squares and circles, respectively. Filled symbols denote the affected participants. The crossed shapes show the deceased individuals. The symbols labeled with asterisks designate the participants of the study. The genotypes are shown below each participant’s symbol. (**ii**) **a**–**e** The asymptomatic presentation of the mother of the affected individuals. **f** Facial picture of patient III-1 showing no facial dysmorphism. **g**, **h** Bilateral ectrodactyly of both hands and unilateral ectrodactyly in the right foot of patient III-1. **i**, **j** Radiographs of the hands and feet, respectively, of the patient (III-1). **k** Facial photograph of patient III-3 with no dysmorphic features. **l**, **m** Unilateral ectrodactyly of the right hand; however, there was no ectrodactyly in her feet. **n**, **o** Radiographs of the hands and feet, respectively. (**iii**) **A** The typical *UBA2* gene structure consists of 17 exons. The variant is located in exon 11. **B** Sanger sequencing chromatograms of the unaffected father (II-1), asymptomatic heterozygous mother (II-2), an affected individual (III-1), an unaffected brother (III-2), and another affected member (III-3) in the pedigree.

Herein, we report the clinical genetic analysis of a Pakistani family with two affected sisters with ectrodactyly/SHFM and variable types of other skeletal anomalies, including clinodactyly and brachydactyly. Peripheral whole blood samples were obtained after informed consent from two affected (III-1 and III-3) and one unaffected siblings (III-2) and both parents (II-1 and II-2; Fig. 1i). The Institutional Review Board (IRB) and Ethics Committees of The University of Central Punjab, Lahore, Pakistan approved the current study. The mother (II-2) of the female patients (III-1, III-3) was phenotypically asymptomatic (Fig. 1ii a–e). The affected girl (III-1) was 25 years old at the time of the study. She presented bilateral ectrodactyly of both hands and unilateral ectrodactyly in the right foot. Photographs and radiographs revealed an aplasia (absence) of the third finger of the left hand and complete aplasia of the third and fourth fingers with metacarpals of the right hand. Additionally, a radiograph of the left hand showed a small hypoplastic bone at the fourth metacarpal, and the second and third metacarpals were fused. In addition, clinodactyly of the second finger of the left hand could be seen in the radiographs. Furthermore, unilateral ectrodactyly in the right foot in the form of complete aplasia of the middle toe with an absence of the central metatarsal as well as a partial fusion of the fourth and fifth metatarsal bones were observed for the right foot (Fig. 1ii, f–j). The second affected girl (III-3) was 27 years old at the time of this study and presented with unilateral ectrodactyly of the right hand. However, she had no ectrodactyly in her feet. Photographs and radiographs revealed polydactyly with syndactyly in the right hand and clinodactyly of the fourth and fifth fingers of the right hand and the third and fifth fingers of the left hand. In addition, the middle finger of the left hand was short, with an absent distal phalanx. Moreover, aplasia of the terminal phalanx of the third toe of the right foot and deviated toes of both feet were notable; however, it was without dislocation (Fig. 1ii, k–o).

Exome sequencing of the DNA from the affected sisters was performed to identify the causal genetic variant. Exome sequencing, filtering strategy and validation/cosegregation of the candidate variants are described previously elsewhere^3^. Upon analysis, we identified a heterozygous 1-bp deletion (NC_000019.9 (NM_005499.3): c.1118del) in exon 11 of the *UBA2* gene residing on chromosome 19q13.11 (Fig. 1iii A). The variant is predicted to affect the frameshift of the *UBA2* gene by creating a premature stop codon (NM_005499.3 (NP_005490.1):p.(Arg373Asnfs*21) and may lead to nonsense-mediated decay (NMD) of the truncated transcripts. Both affected sisters (III-1 and III-3) were heterozygous (WT/Mut) for this 1-bp deletion (Fig. 1iii B III-1, III-3). It was absent in the unaffected father (II-1) and unaffected sibling (III-2) (Fig. 1iii B II-1, III-2); however, the phenotypically unaffected mother (II-2) was heterozygous for this deletion (Fig. 1iii B II-2). The absence of ectrodactyly/SHFM and other clinical symptoms associated with UBA2 mutations in the unaffected mother may be due to incomplete penetrance, especially in autosomal dominant genetic disorders. Patients harboring a disease-causing variant remain asymptomatic throughout their life. According to ACMG and AMP guidelines^4^, the *UBA2* variant is predicted to be pathogenic (PVS1, PM2, PP1, PP4), with a CADD score of 25.5. Additionally, this variant is absent in gnomAD. *UBA2* had a high pLI (1.0) score (probability of loss-of-function intolerance). The pLI score ranges from 0 (most tolerant) to 1 (most intolerant) for genes containing loss-of-function mutations^5^.

The affected individuals in this report showed significant phenotypic variability and reduced penetrance. This is in line with typical SHFM features, as demonstrated by individuals with chromosome 19q13.11 microdeletion syndrome (encompassing the *UBA2* gene) and point mutations in the *UBA2* gene^6–8^. UBA2 (ubiquitin-like modifier-activating enzyme 2, OMIM *613295) is highly expressed in the developing limb buds of mice^9^. The UBA2 ortholog (uba2) in zebrafish was found to be expressed in the eye, brain, and pectoral fins. Furthermore, uba2-null fish were found to have low growth, microphthalmia, microcephaly, abnormal fins, and mandibular hypoplasia^6^. *UBA2* is composed of 17 coding exons and encodes a protein of 640 amino acids. According to HGMD® Professional 2022.2, there are only five reports of 15 pathogenic variants in *UBA2*. Recently, a heterozygous frameshift (c.327delT, p. Phe109Leufs*3) variant in *UBA2* was reported by Wang et al. in a boy and his mother. The affected boy had aplasia cutis congenita, bilateral SHFM/ectrodactyly, and other anomalies. However, the mother, also heterozygous for the same variant, had aplasia cutis congenita but was otherwise healthy, suggesting incomplete or reduced penetrance^10^. In conclusion, the current study reports a novel frameshift variant in *UBA2* in a Pakistani family with SHFM. Our findings extend the phenotypic spectrum and allelic heterogeneity of UBA2-related phenotypes and further emphasize the vital role of *UBA2* in limb development.

## Data

The variant data are submitted to ClinVar, and an accession number (SCV002754416) has been assigned.

## Data Availability

The relevant data from this Data Report are hosted at the Human Genome Variation Database at 10.6084/m9.figshare.hgv.3292.
